# Characterization of biotinylated human ACE2 and SARS-CoV-2 Omicron BA.4/5 spike protein reference materials

**DOI:** 10.1007/s00216-024-05413-7

**Published:** 2024-06-28

**Authors:** Bradley B. Stocks, Marie-Pier Thibeault, Denis L’Abbé, Muhammad Umer, Yali Liu, Matthew Stuible, Yves Durocher, Jeremy E. Melanson

**Affiliations:** 1 Metrology, National Research Council Canada, 1200 Montreal Road, Ottawa, ON K1A 0R6 Canada; 2 Human Health Therapeutics, National Research Council Canada, 6100 Royalmount Avenue, Montreal, QC H4P 2R2 Canada

**Keywords:** COVID-19, Omicron spike protein, ACE2, Reference materials, Binding affinity

## Abstract

Accurate diagnostic and serology assays are required for the continued management of the COVID-19 pandemic yet spike protein mutations and intellectual property concerns with antigens and antibodies used in various test kits render comparability assessments difficult. As the use of common, well-characterized reagents can help address this lack of standardization, the National Research Council Canada has produced two protein reference materials (RMs) for use in SARS-CoV-2 serology assays: biotinylated human angiotensin-converting enzyme 2 RM, ACE2-1, and SARS-CoV-2 Omicron BA.4/5 spike protein RM, OMIC-1. Reference values were assigned through a combination of amino acid analysis via isotope dilution liquid chromatography tandem mass spectrometry following acid hydrolysis, and ultraviolet–visible (UV–Vis) spectrophotometry at 280 nm. Vial-to-vial homogeneity was established using UV–Vis measurements, and protein oligomeric status, monitored by size exclusion liquid chromatography (LC-SEC), was used to evaluate transportation, storage, and freeze–thaw stabilities. The molar protein concentration in ACE2-1 was 25.3 ± 1.7 µmol L^−1^ (*k* = 2, 95% CI) and consisted almost exclusively (98%) of monomeric ACE2, while OMIC-1 contained 5.4 ± 0.5 µmol L^−1^ (*k* = 2) spike protein in a mostly (82%) trimeric form. Glycoprotein molar mass determination by LC-SEC with multi-angle light scattering detection facilitated calculation of corresponding mass concentrations. To confirm protein functionality, the binding of OMIC-1 to immobilized ACE2-1 was investigated with surface plasmon resonance and the resulting dissociation constant, *K*_D_ ~ 4.4 nM, was consistent with literature values.

**Graphical Abstract:**

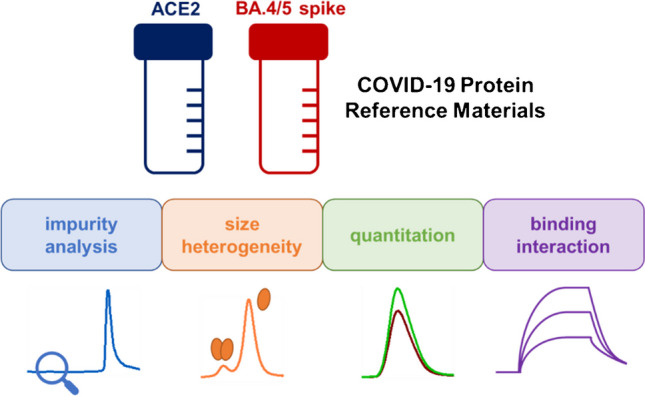

**Supplementary Information:**

The online version contains supplementary material available at 10.1007/s00216-024-05413-7.

## Introduction

The COVID-19 pandemic persists with the continued genetic evolution of the causative SARS-CoV-2 virus. Early variants-of-concern produced distinct infection waves in Canada and elsewhere, yet the Omicron variant has endured and its multiple subvariants continue to dominate [1]. The surface of SARS-CoV-2 is decorated with spike protein and host infection is facilitated through the spike receptor-binding domain (RBD) interacting with its cell-surface receptor, angiotensin-converting enzyme 2 (ACE2) [2]. Consequently, vaccination strategies predominantly endeavor to induce an immune response to the spike protein in hopes of producing neutralizing antibodies (Abs) capable of abrogating spike-ACE2 binding. Early vaccine formulations targeted the spike protein of the ancestral (Wuhan-Hu-1) strain; however, the numerous mutations within the Omicron spike RBD [3] facilitate antibody escape from both vaccine and convalescent serum [4], spurring newer bivalent and subvariant-specific vaccine iterations [5].

In addition to immune evasion, increased viral fitness can be conferred through mutation via enhanced interaction with the target receptor. Spike binds ACE2 with high affinity and Omicron variants exhibit even tighter interactions than the ancestral strain [6] — altered trimer conformations with more accessible RBDs [7–10], and increased positive surface charge on the RBD [11] are potential contributors. Additional studies have demonstrated the contributions of individual, and multiple concurrent, RBD mutations to either increased ACE2 affinity or immune evasion [12–14]. While variant-specific changes in the spike protein amino acid sequence are crucial to ACE2 binding, the interaction is also mediated in part through surface glycan moieties [15, 16]. Each spike monomer contains 22 N-glycosylation sites while ACE2 contains seven, and considerable efforts have revealed near-complete site occupancy and extensive within-site glycan heterogeneity for both spike [15, 17] and ACE2 [16, 18]. Variable spike glycosylation states exhibit differential infectivity [19] while alterations to glycan composition modulate receptor binding and susceptibility to neutralizing antibodies [20]. Indeed, the RBD mutations coupled with its altered glycan profile increase the surface accessibility of Omicron spike protein compared to the ancestral version [21]. The combination of spike mutations that impact ACE2 and antibody binding directly by altering protein structure, along with indirect effects such as mutation-induced changes in glycosylation, explains why some serology assays developed for the ancestral spike protein may exhibit reduced sensitivity to Omicron variants [22].

SARS-CoV-2 serological assay development has proceeded with unprecedented pace, yet rigorous quality assessments often lag behind manufacturing [23]. Accurate diagnostic and surveillance assays are critical to ongoing pandemic management efforts and reference materials (RMs) promote measurement accuracy and reliability through common, well-characterized reagents [24, 25]. Recent studies employing SARS-CoV-2 nucleocapsid protein RMs revealed dramatic differences in the analytical sensitivities of approved rapid antigen detection kits [26, 27], and analogous variability amongst manufacturers has been demonstrated on anti-SARS-CoV-2 antibody kits [28], potentially due to differences in antigens and antibodies. Notably, the use of standardized protein materials and protocols in a recent multi-site serology study produced highly consistent results [29]. We have previously produced an ancestral strain spike protein reference material (RM) [25]; however, as noted above, the sensitivity of some established serological assays may suffer when challenged with Omicron variants [22]. To facilitate continued COVID-19 measurement accuracy and standardization, we have developed both Omicron BA.4/5 spike and biotinylated human ACE2 protein RMs. The production and characterization of these materials are predicted to increase confidence and harmonization in SARS-CoV-2 immunological testing results.

## Materials and methods

### Chemicals and standards

NRC amino acid certified reference materials ALEU-1 (leucine), APHE-1 (phenylalanine), and APRO-1 (proline) were used as SI-traceable primary standards for amino acid analysis (AAA). The corresponding isotope-labelled amino acids were purchased from Cambridge Isotope Laboratories (Tewksbury, MA): [^13^C_6_]-leucine, [ring-^13^C_6_]-phenylalanine, and [^13^C_5_]-proline. Recombinant humanized IgG1κ monoclonal antibody reference material (NISTmAb, RM 8671) was obtained from NIST (Gaithersburg, MD). AAA-grade 6 M hydrochloric acid, Dulbecco’s phosphate-buffered saline (DPBS), EDTA, bovine serum albumin, and chymotrypsin were from Sigma (Oakville, ON). Acetonitrile, formic acid, ammonium acetate, and Tween-20 were purchased from Fisher Scientific (Nepean, ON). Glacial acetic acid was from ACP Chemicals (Montreal, QC), HEPES sodium salt was obtained from Bioshop (Burlington, ON), and sequencing grade trypsin was from Promega (Madison, WI).

### Protein production and reference material preparation

#### ACE2-1

Recombinant, biotinylated human ACE2 protein (amino acids 20–614 of human ACE2 [UniProt Q9BYF1] with N-terminal dualStrep-His_6_-FLAG affinity tags and C-terminal biotin acceptor peptide) was produced and purified as described previously [29] but with an additional in vitro biotinylation step. The full amino acid sequence of expressed protein is listed in Figure S1. Production was performed using CHO^55E1^™ cells by co-transfecting plasmids encoding the ACE2 construct and BirA biotin ligase. At 7 days post-transfection, cell supernatant was harvested and purified sequentially by IMAC and Strep-affinity chromatography. The purified product was buffer exchanged into bicine buffer (50 mM bicine, 100 mM potassium glutamate, pH 8.3) using Amicon Ultra-15 10-kDa centrifugal filters (Millipore). In vitro biotinylation was performed using BirA protein from MCLAB: 55.8 mg (8.8 mL) of ACE2 protein (purified as described above) was combined with 7.45 mL bicine buffer, 1.83 mL 10 × reaction buffer (supplied with BirA protein) and 0.22 mL (0.22 mg) of BirA protein and incubated for 1 h at 30 °C in a circulating water bath. An additional IMAC purification step was performed to re-purify the ACE2 from BirA and other biotinylation reaction components. The final purified product was buffer exchanged into DPBS using Centripure P100 desalting columns (EMP Biotech) and frozen at —80 °C in 2-mL aliquots. For reference material bottling, the bulk protein solution was thawed in a 25 °C water bath, dispensed in 50-µL aliquots into sterile 0.5-mL cryovials, labelled, and stored at − 80 °C.

#### OMIC-1

Spike protein was produced as described previously [30, 31]. The protein construct consists of the full ectodomain of the BA.4/BA.5 variant SARS-CoV-2 spike protein (including native N-terminal signal peptide) fused with human resistin (to mediate trimerization) at the C-terminus; the full amino acid sequence of expressed protein is listed in Figure S2. We note here the absence of the C-terminal affinity tags present in the sequence of our reference strain spike protein RM. Briefly, a CHO^2353^™ cell pool stably transfected with a pTT241 plasmid encoding the spike protein was induced with cumate during a 10-day fed-batch production process in shake flasks. Harvested cell-free supernatant was purified using an in-house multi-step non-affinity-based proprietary method; proteins purified using this method have been tested extensively and used in several previous studies [7, 32, 33]. Following purification, the protein was formulated in DPBS (Gibco) supplemented with 10 mM HEPES sodium salt using tangential flow filtration (TFF)/diafiltration using Minimate 30 kDa TFF capsules (Pall). To assess final purity, proteins were mixed 3:1 (v/v) with XT sample buffer 4 × (Bio-Rad) with (R, reduced) or without (NR, non-reduced) reducing agent (dithiothreitol, 200 mM) and heat-denatured at 70 °C for 10 min. Three micrograms protein was separated by electrophoresis (40 min, 200 V in Bio-Rad MES Running Buffer) using NuPAGE 4–12% Bis–Tris gels (Invitrogen) followed by Coomassie Blue total protein staining. Bulk purified material was frozen in 50-mL Falcon tubes at − 80 °C. For reference material bottling, the bulk protein solution was thawed in a 25 °C water bath, dispensed in 0.2-mL aliquots into sterile 0.5-mL cryovials, labelled, and stored at − 80 °C.

### Sequence analysis and host-cell protein identification by LC–MS/MS

ACE2-1 and OMIC-1 were reduced and alkylated following standard procedures, and then deglycosylated with PNGase F (New England BioLabs, Ipswich, MA) as per the manufacturer’s instructions. Aliquots were subsequently digested overnight at 37 °C with trypsin (1:20 w/w) or chymotrypsin (1:50 w/w). Peptides were injected onto a 2.1 × 150 mm BEH130 C18 column (Waters, Milford, MA) kept at 30 °C, and eluted via a water:acetonitrile gradient in the presence of 0.1% formic acid produced with a Thermo Fisher Scientific Vanquish UHPLC (San Jose, CA). Solvent composition ramped from 5 to 40% ACN over 23 min, increased to 60% in 5 min, and then was washed with 95% ACN for 4 min prior to a 5-min re-equilibration step at initial conditions. Column eluant was directed into the HESI source of an Orbitrap Fusion Lumos (ThermoFisher Scientific) with an ESI needle voltage of + 3800 V and sheath, auxiliary, and sweep gas flows of 35, 10, and 2, respectively (arbitrary units). The ion transfer tube and vaporizer were heated to 275 and 250 °C, respectively. Survey scans were acquired from *m*/*z* 350–1400 at 60,000 resolution and RF 30% while data-dependent MS/MS proceeded via HCD (30% normalized CE) with fragment ion spectra recorded in the ion trap. Data analysis was performed in MaxQuant 2.3 employing the *Cricetulus griselus* Uniprot reference proteome database supplemented with the ACE2-1 or OMIC-1 sequence, in addition to PNGase F and common contaminants. Proteins detected by MS/MS with at least two unique peptides were considered positive identifications. Host-cell protein abundances were estimated from tryptic digests using riBAQ [34] within MaxQuant and are shown in Tables S1 and S2.

### Amino acid analysis by LC-ID-MS/MS

Isotopically labelled amino acid (^13^C_6_-Leu, ^13^C_6_-Phe, and ^13^C_5_-Pro) stock solutions were prepared gravimetrically in ultrapure water, and then combined as internal standard in mole fractions representing those in the ACE2-1 (or OMIC-1) sequence to enable exact-matching isotope dilution (1:1 ratio). A corresponding solution of the natural amino acids (Leu, Phe, and Pro), and two blends bracketing the 1:1 ratio (0.5 and 1.5 ratio) were prepared as calibrants [35]. Equal amounts of the internal standard were then spiked into each of the calibration blends and ACE2-1 (or OMIC-1), followed by liquid-phase acid hydrolysis in 6 M HCl. The solutions were incubated at 110 °C for 72 h to ensure complete peptide bond cleavage and then equilibrated to ambient temperature. A 40-μL aliquot of each solution was diluted to 1 mL prior to LC–MS/MS analysis [36]. NRC SARS-CoV-2 (reference strain) spike protein RM SMT1-1 [25] was spiked with ^13^C-labelled amino acids and hydrolyzed in parallel as a quality control sample.

Hydrolysate separation by LC proceeded as described previously [36]. Briefly, we employed a Macherey–Nagel Nucleodur HILIC column (2.0 × 100 mm, 3.0-µm particle) connected to an Ultimate 3000 UHPLC (ThermoFisher Scientific). For each sample, 4 μL was injected onto the column, which was heated to 35 °C. Isocratic elution proceeded at 0.25 mL min^−1^ with 10 mM ammonium acetate in 88% acetonitrile, adjusted to pH 3.5 with glacial acetic acid. Eluting amino acids flowed into the heated electrospray source of a Thermo Quantiva triple quadrupole MS operating in multiple reaction monitoring (MRM) mode. Compound-specific parameters such as collision energy and dwell time were optimized as reported previously [25]. The ESI needle voltage was set to + 4400 V and the sheath, auxiliary, and sweep gas flows were 25, 17, and 1, respectively (arbitrary units). The ion transfer tube and vaporizer temperatures were set to 325 and 280 °C, respectively. Peak areas were extracted using Xcalibur software from the instrument manufacturer. Quantitation of each amino acid in the sample was performed using the quadruple isotope dilution equation described in Pagliano et al. [37].

### Ultraviolet–visible spectrophotometry

Protein concentrations in ACE2-1 and OMIC-1 were determined using a NanoDrop One^c^ (ThermoFisher Scientific) UV–Visible spectrophotometer at 280 nm. Instrument performance was verified through analysis of NISTmAb RM 8671 and Pierce bovine serum albumin standard. The instrument was blanked with the appropriate RM formulation buffer. Two microliters protein solution was pipetted onto the pedestal and the pathlength was set to 1 mm. Pathlength uncertainty was determined through analysis of Thermo Fisher PV-1 solution. Molar extinction coefficients were predicted from the protein construct amino acid sequences using the ExPASy ProtParam online tool (https://web.expasy.org/protparam): 170 085 M^−1^ cm^−1^ for ACE2-1 (including 3 disulfide bonds) and 141 720 M^−1^ cm^−1^ for OMIC-1 (18 disulfides).

### Size exclusion chromatography

Liquid chromatography was performed on a Waters Acquity UPLC equipped with an extended wavelength photodiode array detector operating at 280 nm, and an autosampler maintained at 10 °C. Instrument and column performance were confirmed through a combination of protein molecular weight standard mix (Waters), NISTmAb RM 8671, and NRC RM SMT1-1. Five microliters ACE2-1 (~ 10 µg ACE2) or 10 µL OMIC-1 (~ 7 µg spike protein) was injected onto a Waters BEH SEC 4.6 × 150 mm column (200 Å or 450 Å pore size for ACE2-1 and OMIC-1, respectively) and eluted at 0.4 mL min^−1^ with DPBS supplemented with 0.02% Tween-20, pH 7.4. Data analysis was done with MassLynx software provided by the instrument manufacturer. Multi-angle light scattering (MALS) and refractive index (RI) data were collected using miniDAWN and Optilab T-rEX detectors, respectively (Wyatt, Santa Barbara, CA), and data were analyzed using the accompanying Astra software (version 8.0.0.28).

### Surface plasmon resonance

SPR data were collected on an ezSPR benchtop instrument (Affinité Instruments, Montreal, QC). Running buffer (RB) was composed of 10 mM HEPES (pH 7–7.4), 150 mM NaCl, 3 mM EDTA, and 0.05% Tween-20. SPR sensor was constructed from a biotin-coated Au SPR chip (Affinité) through successive immobilization of streptavidin (25 µg mL^−1^, 20-min exposure, 1800–2000 RU) and ACE2-1 (2 µg mL^−1^, 15–20-min exposure, 250–300 RU). Sensor was subsequently exposed to 10 µM bovine serum albumin for 20 min to reduce nonspecific analyte binding. A stable baseline was established with RB prior to streptavidin immobilization, and the sensor surface was washed with RB between each step. The sensor was then conditioned by flowing RB over the surface at 50 µL min^−1^ until a stable signal was achieved. Subsequently, a twofold dilution series of analyte (SMT1-1 or OMIC-1) ranging between 12.5 and 200 nM was injected at 50 µL min^−1^ for approximately 90 s to facilitate spike protein association with immobilized ACE2. Given the strong binding affinity of spike trimers with ACE2 [2], dissociation proceeded for 30–45 min at each analyte concentration. Baseline signal recorded during a 5-min window immediately preceding analyte injection was used for drift correction. Raw data was drift corrected using the open-source R-based Anabel application [38]. Drift-corrected binding curves were analyzed with TraceDrawer (version 9.1.2, Ridgeview Instruments), and kinetic parameters for interaction of trimeric SARS-CoV-2 spike variants with ACE2 were calculated using a 1:1 binding model. Reported data are averages from two independent experiments per spike variant.

## Results and discussion

### Protein identity

The purity of bulk protein materials used for the reference materials was first visualized with SDS gel electrophoresis (Figure S3). Mass spectrometry-based peptide mapping experiments indicated > 96% sequence coverage for both ACE2-1 (Figure S1) and OMIC-1 (Figure S2). Total sequence coverage was determined through merging results of separate trypsin and chymotrypsin digests. To maximize coverage around glycan attachment sites, both proteins were deglycosylated with PNGase F prior to proteolytic digestion. The ACE2 protein construct employed a C-terminal recognition sequence which was biotinylated via the *E. coli* biotin ligase BirA. The biotin handle can be exploited for ACE2 immobilization or for binding reporter molecules in protein-based surrogate neutralization assays [39], and near-complete (> 99%) labeling was confirmed with SDS-PAGE and LC–MS/MS (Figure S4).

### Homogeneity

**Fig. 1:**
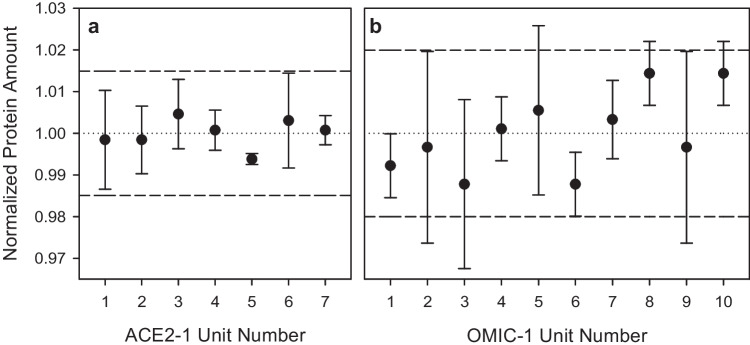
Normalized protein content in randomly selected (**a**) ACE2-1 and (**b**) OMIC-1 units measured by UV–Vis spectrophotometry at 280 nm. Data points and error bars represent averages and standard deviations of triplicate technical replicates. Dotted lines indicate the average molar protein concentration determined by *A*_280_ and dashed lines represent two standard deviations of the mean

To assess vial-to-vial protein content consistency, seven (ACE2-1) and ten (OMIC-1) units were randomly selected from the fill batches of 440 and 600 units, respectively, and molar protein concentration was determined from triplicate UV–vis absorbance measurements at 280 nm (Fig. 1). The between-unit variabilities were calculated using the Bayesian ANOVA embedded within the online NRC CRM homogeneity uncertainty calculator tool (htts://metrology.shinyapps.io/homogeneity-calculator). The model was fit to data using Monte Carlo with R package *rjags*, resulting in homogeneity uncertainties (*u*_hom_, 95% CI) of 0.043 µmol L^−1^ and 0.024 µmol L^−1^ for ACE2-1 and OMIC-1, respectively. Following ISO Guide 35 on the preparation of reference materials [40], the uncertainties due to inhomogeneity were considered negligible in proportion to the respective characterization uncertainties of ACE2-1 (0.738 µmol L^−1^) and OMIC-1 (0.198 µmol L^−1^); therefore, the protein content of both materials was determined to be homogeneous.

**Fig. 2:**
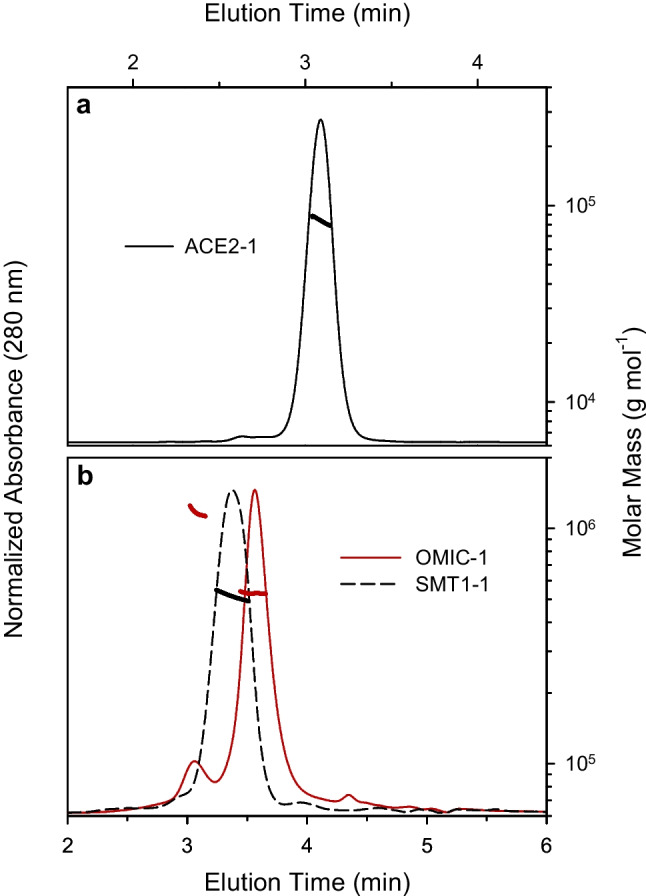
Size exclusion UV chromatograms of (**a**) ACE2-1 and (**b**) OMIC-1 (red line). Also included in (b) is the chromatogram for SMT1-1 (black dashed line). Associated molar mass values (filled circles, right axes) from MALS detection are overlaid. Note that chromatograms in (a) and (b) were generated using SEC columns with difference pore sizes and are thus not directly comparable

In addition to ensuring consistent protein concentration across the fill series, the maintenance of native higher-order structure must be considered for protein RMs. Human ACE2 is expressed on the surface of epithelial cells as a monomer while the SARS-CoV-2 spike protein assembles into a trimer held together by non-covalent interactions. Notably, the presence of human resistin at the C-terminus of the spike protein in OMIC-1 contributes to maintaining the trimeric form [30], which influences the exposed epitopes for potential anti-spike antibodies [7, 41]. As such, alterations to the oligomeric status may affect the results of some assays; therefore, the relative amounts of ACE2 monomers and spike trimers were quantified in ACE2-1 and OMIC-1, respectively, by LC-SEC-UV by comparing elution times to protein size standards. The OMIC-1 measurements were conducted on the same ten units mentioned above whereas the limited volume present in ACE2-1 necessitated an alternative approach. Instead, the oligomeric status was determined using the − 80 °C control samples from the short-term (4 units) and freeze–thaw (3 units) stability studies. The SEC-UV chromatogram shown in Fig. 2a revealed that the ACE2 protein in the ACE2-1 RM is highly monomeric (~ 98%), with the small remainder likely present as dimers. The spike protein in OMIC-1 exhibited more size heterogeneity (Fig. 2b). While the majority (82%) assembled into the native trimer, a significant proportion formed higher molecular weight oligomers (14%) with the remainder present as a low molecular weight species, similar to the distributions observed previously by our group for spike proteins of other SARS-CoV-2 variants [7]. Further, we demonstrated that dilution (2–100 ×) with formulation buffer had no discernible impact on the relative amount of spike protein trimer in OMIC-1 (Figure S5). Finally, no significant between-unit differences in protein size heterogeneity were observed for either RM (Figure S6), providing further evidence of homogeneity.

### Molar mass determination

Both ACE2 and SARS-CoV-2 spike proteins are extensively post-translationally modified — ACE2 contains seven glycosylation sites [42] while each spike monomer contains 22 [17]. Notably, the ACE2 ectodomain construct used to produce ACE2-1 contains only six of the sites as Asn690 is absent (Figure S2). Such modifications are known to cause protein molar mass overestimations in SEC-UV experiments [43] and glycosylation heterogeneity (both macro and micro) often precludes direct intact mass determination with mass spectrometry, although charge detection MS has recently shown promise in this area [44]. Alternatively, SEC coupled to multi-angle light scattering (MALS) detection can facilitate accurate protein mass determinations even for extensively glycosylated analytes [45]; therefore, it was employed to assess both RMs (Fig. 2).

Using SEC-MALS, we determined the ACE2 monomer molar mass to be approximately 84 kDa — the implied glycan contribution of ~ 8 kDa being consistent with multiple bottom-up glycoproteomics studies [15, 42, 46]. The dominant spike protein species observed in the OMIC-1 chromatogram had a molar mass of approximately 525 kDa, consistent with a trimeric assembly. The calculated glycan contribution of ~ 105 kDa was in agreement with previous studies on the reference strain (Wuhan-Hu-1) spike trimer [17, 44, 47], as well as our own reference strain spike protein reference material, SMT1-1 [25]. The MALS data also suggest that the high molecular weight species in OMIC-1 consists of a hexameric form, likely through a dimerization of trimers. The reference strain spike migrated through the SEC column more quickly than the BA.4/5 spike protein, although MALS detection indicated highly consistent molar mass values for the respective trimers (Fig. 2b). Recent hydrogen–deuterium exchange experiments demonstrate that the canonical prefusion compact spike trimer is in equilibrium with a more dynamic open conformation [41], and the reversible interconversion is influenced by not only solution conditions but also variant-specific mutations [7]. An expanded trimer conformation with minimal inter-protomer interactions and held together mainly by the trimerization domain is favored by the reference strain spike protein, whereas a compact trimer with tight association of the spike protein protomers is favored by most other variants, including Omicron [7]. These recent findings help to explain the distinct SEC elution profiles observed here for OMIC-1 and SMT1-1 despite the similarity in their molar mass values.

### Stability

As discussed above, ACE2 receptor and SARS-CoV-2 spike protein are expressed as a monomer and trimer, respectively, and non-native oligomeric forms may occlude (or expose) potential epitopes for antibody binding in serological assays. We therefore monitored the stability of the oligomeric distributions shown in Fig. 2 in response to various stresses: (1) short-term exposure to potential transportation and manipulation conditions, (2) long-term freezer storage, and (3) multiple freeze–thaw cycles.

**Fig. 3:**
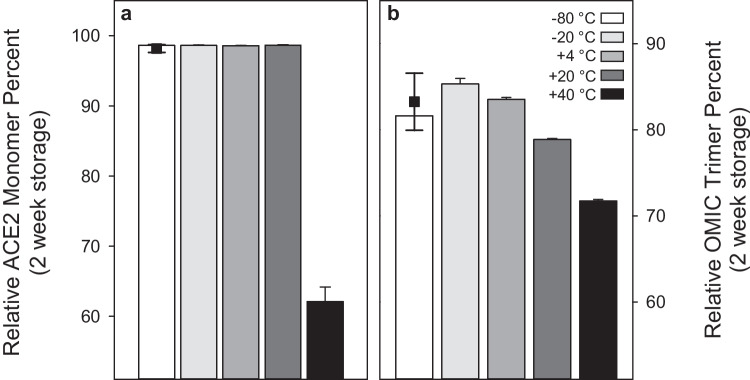
Relative ACE2 monomer percent in ACE2-1 (**a**) and spike trimer percent in OMIC-1 (**b**) measured by LC-SEC-UV after 2-week storage at various temperatures. Bar plots are averages of two units per condition measured in technical triplicate and error bars represent standard deviations. The single data points (black squares) show the average and standard deviation of all − 80 °C samples analyzed across the respective ACE2-1 or OMIC-1 measurement campaigns (value assignment, short-term stability, freeze–thaw stability)

The short-term stabilities of both materials were assessed using identical isochronous approaches, where duplicate samples were incubated at various common laboratory temperatures (− 20, + 4, + 20, and + 40 °C) for various durations (1, 7, and 14 days). All samples were analyzed with SEC-UV with reference to samples kept at − 80 °C. Figure 3 depicts the results for 14-day storage at all temperatures and results from each temperature-duration storage combination for both RMs are shown in Figure S7. Figure 3a shows that only exposure to the most elevated temperature condition caused a decrease in the relative percent of ACE2 monomer in ACE2-1, and the SEC chromatogram revealed this to be caused by higher-order oligomer formation (Figure S8). A similar trend was observed for the spike protein trimer in OMIC-1 (Fig. 3b), where a significant decrease was observed after storage at + 40 °C. Storage at intermediate temperatures had small effects on the relative trimer percent; however, in most cases, the changes were within uncertainty discerned for the − 80 °C reference samples. Similar to ACE2-1, OMIC-1 exposure to elevated temperature caused an increase to the high molecular weight species. Interestingly, the small increase in trimer percent observed upon storage at − 20 °C resulted from a broadening of the SEC elution peak, suggestive of a more open trimer conformation potentially due to a reduction in inter-protomer contacts [7].

**Fig. 4:**
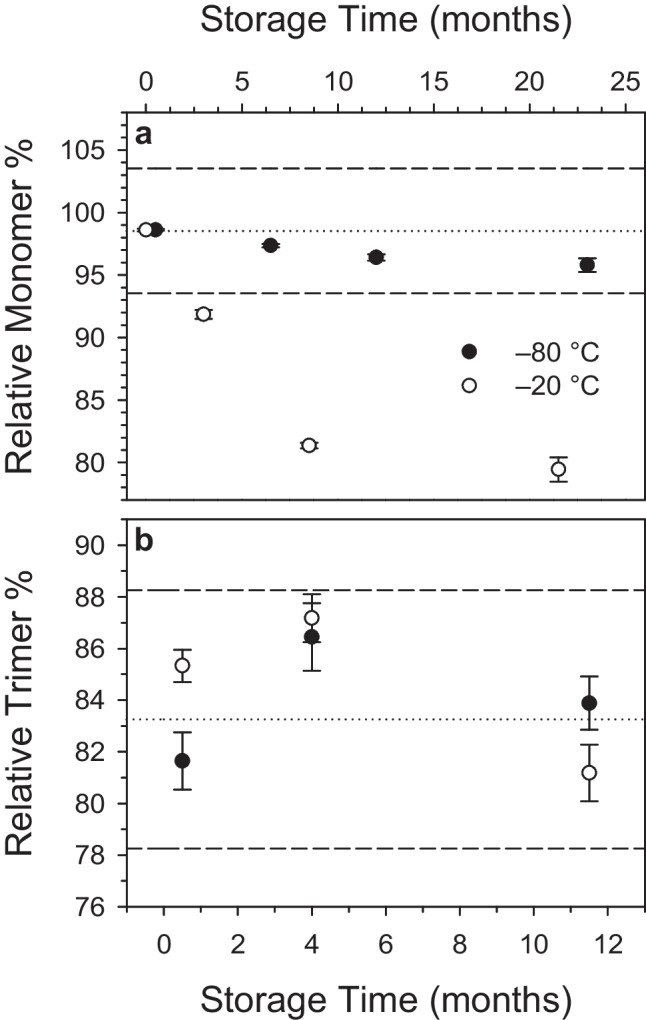
Relative ACE2 monomer percent in ACE2-1 units (**a**) and spike trimer percent in OMIC-1 units (**b**) stored at − 80 °C (black circles) and − 20 °C (white circles). Data points are averages of duplicate units measured in triplicate and error bars represent standard deviations. Dotted lines are the average of all − 80 °C samples measured across the RM characterization campaigns (value assignment, short-term and freeze–thaw stability). The dashed lines show ± 5% and are for visualization purposes only. Note that in panel (a), the 0.5-month data point for − 20 °C has been shifted by − 0.4 months to reduce overlap

Prolonged storage of protein solutions often necessitates freezer conditions; thus, the long-term stabilities of ACE2-1 and OMIC-1 held at − 20 and − 80 °C were investigated. A small (< 3%) decrease in ACE2 monomer was measured after 2 years at − 80 °C (Fig. 4a); however, significant losses were observed from units stored at − 20 °C for only 6 months, with further time-dependent decreases up to 23 months. This observed SEC-UV result correlates with previous work showing loss of serum ACE2 activity after extended storage at − 20 °C [48]. In contrast to ACE2-1, OMIC-1 displayed no significant change in its size distribution after 1 year when stored at either − 20 or − 80 °C (Fig. 4b) mirroring the long-term freezer stability of our reference strain spike protein reference material SMT1-1 [25]. In addition to the effects of long-term storage on size heterogeneity, we assessed protein concentration via UV–vis after extended storage at − 80 °C and detected no significant differences in either ACE2-1 (after 2 years) or OMIC-1 (1 year) (Figure S9).

The stabilities of ACE2-1 and OMIC-1 were evaluated using all respective short-term and long-term measurements. We again employed Bayesian ANOVA, along with the Arrhenius equation to link the rate constants measured at the various storage temperatures, via the NRC CRM stability uncertainty online calculator (https://metrology.shinyapps.io/stability-calculator). The fitted models were then used to predict potential RM degradation at + 40 °C for 2 days, conditions potentially encountered during a significant shipping delay. Additionally, the long-term freezer storage stabilities were predicted for ACE2-1 (− 80 °C for 3 years) and OMIC-1 (− 20 °C for 5 years). Notably, OMIC-1 storage stability was predicted at − 20 °C as this gave a more conservative estimate of potential degradation, whereas significant ACE2-1 degradation at − 20 °C has already been established. The resulting short- and long-term uncertainty components were combined into the final stability uncertainties for the two protein RMs (Table 1).

**Table 1:** Reference value uncertainty components for ACE2-1 and OMIC-1

Substance	*U*_*k*=2_	*u*_c_	*u*_char_	*u*_hom_	*u*_stab_
Angiotensin-converting enzyme 2 molar concentration (µmol L^−1^)	1.7	0.9	0.7	0.0	0.5
Angiotensin-converting enzyme 2 mass concentration (mg mL^−1^)	0.13	0.065	0.055	0.00	0.034
Angiotensin-converting enzyme 2 glycoprotein mass concentration (mg mL^−1^)	0.2	0.094	0.087	0.00	0.035
SARS-CoV-2 BA.4/5 spike protein molar concentration (µmol L^−1^)	0.5	0.25	0.2	0.0	0.15
SARS-CoV-2 BA.4/5 spike protein mass concentration (mg mL^−1^)	0.07	0.035	0.028	0.00	0.022
SARS-CoV-2 BA.4/5 spike glycoprotein mass concentration (mg mL^−1^)	0.09	0.05	0.04	0.00	0.03

**Fig. 5:**
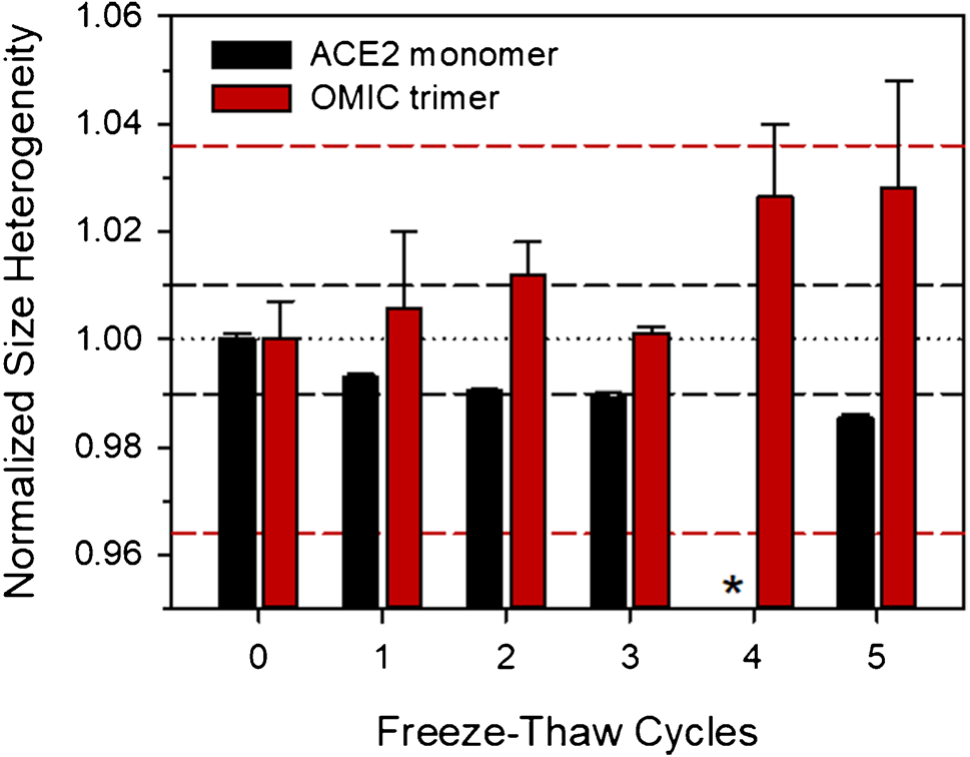
Freeze–thaw effects on protein size heterogeneity. Relative percent of ACE2 monomer in ACE2-1 (black bar) and spike trimer in OMIC-1 (red bar) was measured after repeated freeze–thaw cycles and normalized to a control sample (zero cycles). Bars are averages of duplicate vials measured in technical triplicate and error bars represent standard deviations. Dashed lines indicate combined standard uncertainty of all − 80 °C control measurements from homogeneity, stability, and freeze–thaw studies for ACE2-1 (1.0%, black dash) and OMIC-1 (3.6%, red dash). Samples subjected to four freeze–thaw cycles were not evaluated for ACE2-1 (denoted by asterisk)

Finally, the stabilities of ACE2-1 and OMIC-1 over one to five freeze–thaw (F/T) cycles were investigated. Figure 5 shows that no significant large-scale changes to either protein were measured as a result of F/T; however, small general trends (ACE2 monomer decrease, spike trimer increase) were observed with increasing cycle number. While no explicit F/T uncertainty components were included in the final budgets, recommendations for minimizing F/T cycles were included in the accompanying certificates for both materials.

### Reference value assignment and uncertainty evaluation

ACE2-1 and OMIC-1 are reference materials evaluated for ACE2 and spike protein content, respectively. The molar protein concentration of each material was calculated as the average of values determined by two orthogonal methods — amino acid isotope dilution mass spectrometry following acid hydrolysis, and UV–vis spectrophotometry. Aliquots required for both methods were successively sampled from seven ACE2-1 or ten OMIC-1 units to avoid potential freeze–thaw effects. Absorbance readings at 280 nm were measured directly on a microvolume spectrophotometer with the pathlength set to 0.1 cm and were converted to protein concentrations using the molar extinction coefficients calculated from the amino acid sequences. The combined measurement uncertainty included the standard deviation of triplicate measurements and the pathlength uncertainty determined during instrument verification (Table S1). Extinction coefficients were calculated as per Pace et al. [49], and the median relative difference between theoretical and experimental coefficients for the 80 proteins investigated therein (2.9%) was used here as the corresponding uncertainty component. Employing the molar rather than the mass extinction coefficient avoids additional uncertainty over the appropriate molar mass value, i.e., protein sequence with or without the attached glycans. The average protein concentration and combined standard uncertainty (*k* = 1) were 25.5 ± 0.9 µmol L^−1^ for ACE2-1, and 5.3 ± 0.2 µmol L^−1^ for OMIC-1. Excellent agreement was obtained with amino acid analysis ID-MS (ACE2-1, 25.2 ± 0.5 µmol L^−1^; OMIC-1, 5.4 ± 0.2 µmol L^−1^) where the uncertainty values were most heavily influenced by the LC–MS isotope ratio measurement repeatability (Figure S10). Due to the lack of an explicit impurity correction and the incomplete understanding of the uncertainty associated with the UV–vis method, we opted for a conservative data combination approach. For each reference material, the consensus molar concentration was determined as the arithmetic average of the two values, and the resultant uncertainty was combined with those from the short- and long-term stability studies (Tables 1 and 2) as the root sum squared. While the molar protein concentration does not depend on the glycosylation status, the more frequently used mass concentration depends on the total molar mass and therefore can differ significantly whether the glycan portion is considered or not. The mass concentrations for both the protein-only and glycoprotein analytes, for both ACE2-1 and OMIC-1, are listed in Table 2 and were calculated using the noted molar mass and formulation buffer density.

**Table 2:** Reference values and expanded uncertainties (*k* = 2, 95% CI) for ACE2-1 and OMIC-1

Substance	Molar concentration (µmol L^−1^)	Mass concentration (mg mL^−1^)^†^
Angiotensin-converting enzyme 2	25.3 ± 1.7	1.9 ± 0.1^a^
Angiotensin-converting enzyme 2 glycoprotein	25.3 ± 1.7	2.1 ± 0.2^b^
SARS-CoV-2 BA.4/5 spike protein	5.4 ± 0.5	0.76 ± 0.07^c^
SARS-CoV-2 BA.4/5 spike glycoprotein	5.4 ± 0.5	0.94 ± 0.09^d^

^†^Calculated using measured RM formulation buffer density, 1.01 ± 0.01 g mL^−1^

^a^Biotinylated ACE2 protein sequence only, 76 107 ± 1 g mol^−1^

^b^Biotinylated ACE2 protein plus best estimate of total glycan mass, 84 000 ± 5000 g mol^−1^

^c^BA.4/5 spike protein sequence only, 140 323 ± 1 g mol^−1^

^d^BA.4/5 spike protein plus best estimate of total glycan mass, 175 000 ± 5000 g mol^−1^

As discussed above in the “Materials and methods” section, the bulk protein material used for both RMs underwent multiple purification steps prior to bottling. Despite this extensive clean-up, small amounts of contaminating host-cell proteins (HCPs) persisted, as has been reported for other protein reference materials [50, 51]. Both UV–Vis and ID-MS report on the total protein amount in solution; therefore, we employed a proteomic approach [34] to identify CHO HCPs and estimate their abundances (Tables S2 and S3). Many fewer proteins were measured in ACE2-1 (4) than in OMIC-1 (48) along with a much lower relative total HCP amount (0.1% vs 2.7%). The difference in HCPs observed here likely results from the use of three affinity purification steps for the ACE2 protein compared to three non-affinity purification steps for the spike protein; however, host-cell protein identification can vary dramatically depending on many experimental parameters [50]. Because of the large uncertainty associated with HCP measurements, we did not explicitly correct the reference values for their presence, rather an uncertainty component equal to their total relative amount was added [36] into the budgets for both UV–vis and ID-MS measurements (Table S3).

### Binding affinity

**Fig. 6:**
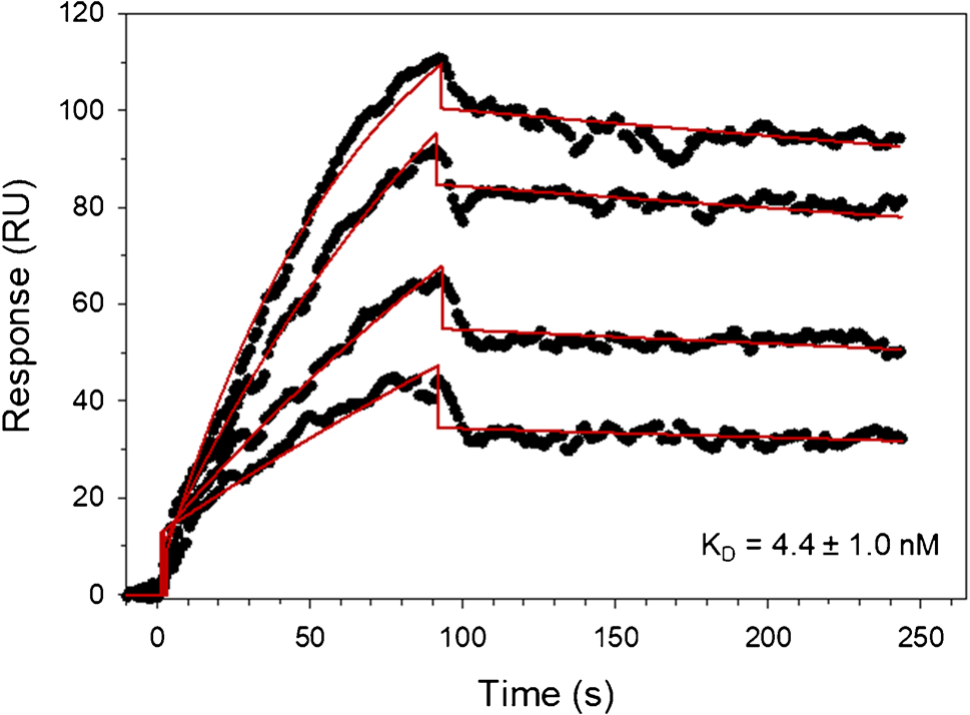
Binding affinity of OMIC-1 for immobilized ACE2-1. Spike protein concentrations covered a range of 12.5–100 nM (black circles). A 1:1 binding model was fit (red lines) to the data and the equilibrium dissociation constant represents the average and standard deviation of duplicate independent measurements

Surface plasmon resonance was employed to determine the affinity and kinetic interaction parameters of spike protein variants with ACE2 receptor. The site-specific biotinylation near the ACE2-1 C-terminus allows for efficient ligand immobilization while retaining proper protein orientation and conformation. The ligand surface density is a critical parameter in SPR assay development, and careful optimization is required for instrument sensitivity and resolution while minimizing steric hindrance and analyte reassociation over a range of concentrations [52]. In our experimental setup, an ACE2-1 ligand density corresponding to an SPR response of 250–300 RU generated reproducible kinetic data over a range of at least four twofold analyte dilutions, while concomitantly avoiding problems such as analyte reassociation or ligand saturation (Figs. 6 and S8). To facilitate comparison with previous studies, in addition to OMIC-1 analytes, we also employed our reference strain spike protein reference material, SMT1-1.

Suitable fits of a 1:1 binding model to the data were achieved for at least four different twofold analyte dilutions across two independent experiments for both OMIC-1 (12.5–100 nM, Fig. 6) and SMT1-1 (25–200 nM, Figure S11). The kinetic constants were determined using the average and standard deviations of the duplicate measurements. The calculated equilibrium dissociation constants (*K*_D_) for the OMIC-1:ACE2-1 and SMT1-1:ACE2-1 interactions were 4.4 ± 1.0 nM and 17.1 ± 1.8 nM, respectively. While the association rate for both spike proteins was similar, the increased affinity observed for the BA.4/5 variant resulted mainly from a decreased dissociation rate (Table 3). The *K*_D_ values measured here are consistent with previous studies [4, 53, 54], and the trend conforms to the literature consensus that Omicron variants bind ACE2 with higher affinity than the ancestral reference strain spike protein [6, 55, 56].

**Table 3:** Kinetic parameters for spike protein reference material interaction with ACE2-1

Spike protein RM	*k*_on_ (× 10^5^ M^−1^ s^−1^)	*k*_off_ (× 10^−3^ s^−1^)	*K*_D_ (nM)
SMT1-1	1.1 ± 0.1	1.8 ± 0.3	17.1 ± 1.8
OMIC-1	1.5 ± 0.1	0.7 ± 0.2	4.4 ± 1.0

## Conclusions

The continued evolution of the SARS-CoV-2 Omicron lineage has allowed the COVID-19 pandemic to persist. mRNA vaccine formulations now undergo regular updates to induce expression of dominant Omicron variant spike proteins, yet similar development and validation of variant-specific serological assays have lagged. Further, comparability between assays remains under-investigated, largely due to the proprietary nature of antibody-antigen pairs employed by individual manufacturers and the lack of suitable protein calibrators. To facilitate such comparability evaluations of serological assays, we have produced and characterized two protein reference materials: human ACE2 (ACE2-1) and Omicron BA.4/5 spike protein (OMIC-1). We note here that as of writing, BA.4/5 is no longer the dominant circulating SARS-CoV-2 strain; however, it displays much greater similarity to more recently dominant Omicron variants (e.g., XBB.1.5) than the ancestral strain. Both reference materials were found to be homogeneous and stable under shipping and storage conditions, in addition to typical laboratory manipulation conditions. Finally, the high binding affinity measured between the two proteins demonstrated their functionality. The two materials are envisioned to be employed as standard protein reagents in the development of SARS-CoV-2 serological tests.

### Supplementary Information

Below is the link to the electronic supplementary material.

Supplementary file1 (DOCX 773 KB) A280 measurement uncertainty budget; CHO host-cell protein identifications; ACE2-1 and OMIC‑1 peptide mapping; SDS-PAGE; ACE2 biotinylation determination; OMIC-1 dilutions by SEC; LC-SEC-UV homogeneity analysis; Short-term stability of ACE2-1 and OMIC-1; Thermally induced aggregation; Long-term storage stability by UV-vis; LC-ID-MS/MS amino acid quantitation; SMT1-1:ACE2-1 binding by SPR
