# Supplementary material for: Characterization of biotinylated human ACE2 and SARS-CoV-2 Omicron BA.4/5 spike protein reference materials
Source: Anal Bioanal Chem. 2024 Jun 28;416(22):4861–72. doi: 10.1007/s00216-024-05413-7 (PMC11330416; doi:10.1007/s00216-024-05413-7)
Supplement: Supplementary file 1 — Supplementary file1 (DOCX 773 KB) A280 measurement uncertainty budget; CHO host-cell protein identifications; ACE2-1 and OMIC‑1 peptide mapping; SDS-PAGE; ACE2 biotinylation determination; OMIC-1 dilutions by SEC; LC-SEC-UV homogeneity analysis; Short-term stability of ACE2-1 and OMIC-1; Thermally induced aggregation; Long-term storage stability by UV-vis; LC-ID-MS/MS amino acid quantitation; SMT1-1:ACE2-1 binding by SPR [file 216_2024_5413_MOESM1_ESM.docx]

*Supplementary Information*

**Characterization of biotinylated human ACE2 receptor and SARS‑CoV-2 omicron BA.4/5 spike protein reference materials**

Bradley B. Stocks^1^*, Marie-Pier Thibeault^1^, Denis L’Abbé^2^, Muhammad Umer^1^, Yali Liu^2^, Matthew Stuible^2^, Yves Durocher^2^, and Jeremy E. Melanson^1^

^1^*Metrology, National Research Council Canada*

*1200 Montreal Road, Ottawa, Ontario, Canada K1A 0R6*

^2^*Human Health Therapeutics, National Research Council Canada*

*6100 Royalmount Avenue, Montreal, Quebec, Canada H4P 2R2*

**Table S1.** UV-Vis A280 uncertainty budget contributions

| **Component** | **ACE2-1 (rel. %)** | **OMIC-1 (rel. %)** |
| --- | --- | --- |
| Standard deviation | 0.74 | 1.0 |
| Pathlength | 1.92 | 0.6 |
| Extinction coefficient | 2.9 | 2.9 |
| Host-cell proteins | 0.1 | 2.7 |
| Combined standard uncertainty (rel. %) | 3.6 | 4.1 |
| Combined standard uncertainty (µmol L^-1^) | 0.93 | 0.22 |

**Table S2.** Host-cell proteins detected in ACE2-1 by LC-MS/MS

| **Accession** | **Protein Name** | **Relative Abundance (µmol/mol ACE2)** |
| --- | --- | --- |
| G3H7B3 | Galectin | 322 |
| G3IFV1 | Centrosomal protein of 135 kDa | 190 |
| G3HZI4 | Golgin subfamily A member 1 | 159 |
| G3GWS7 | A-kinase anchor protein 8-like | 19 |

**Table S3.** Host-cell proteins detected in OMIC-1 by LC-MS/MS

| **Accession** | **Protein Name** | **Relative Abundance (µmol/mol spike)** |
| --- | --- | --- |
| G3H8V5 | Carboxypeptidase | 3274 |
| G3H8Y4 | Collagen alpha-2(VI) chain | 3135 |
| G3I1H5 | Asparaginyl endopeptidase | 3016 |
| G3HNJ3 | Clusterin | 2449 |
| G3H8Y3 | Collagen alpha-2(VI) chain | 2053 |
| G3H8Y5 | Collagen alpha-1(VI) chain | 1800 |
| Q9EPP7 | Cathepsin X | 1664 |
| G3IBH0 | Metalloproteinase inhibitor 1 | 1602 |
| G3GXB0 | ARHGDIA | 1187 |
| G3H0L9 | Cathepsin B | 599 |
| G3HE67 | CREG1 | 484 |
| G3H604 | Alpha-galactosidase | 376 |
| G3I1V3 | Fibronectin | 346 |
| G3INC5 | Cathepsin L1 | 294 |
| G3H1D5 | Carboxypeptidase | 281 |
| G3HRK9 | Matrix metalloproteinase-19 | 271 |
| G3HCS9 | Nucleosome-remodeling factor subunit BPTF | 254 |
| G3HMG4 | Amyloid-beta A4 protein | 202 |
| G3H2W6 | Extracellular matrix protein 1 | 201 |
| G3HS71 | Vasorin | 194 |
| G3I973 | Hypoxia up-regulated protein 1 | 187 |
| G3HEE8 | Alpha-L-iduronidase | 183 |
| G3HZE3 | Exo-alpha-sialidase | 181 |
| G3HGM6 | AGA | 161 |
| G3H8F4 | Alpha-dystroglycan | 150 |
| G3H7I6 | Sulfhydryl oxidase | 147 |
| G3HAI3 | Follistatin-related protein 1 | 135 |
| G3HMV7 | Alpha-L-fucosidase | 128 |
| G3INA5 | Transcription termination factor 1 | 114 |
| G3HQL6 | Thioredoxin reductase 1, cytoplasmic | 111 |
| G3IDE4 | Tripeptidyl-peptidase 1 | 110 |
| G3HUI4 | Lysosomal Pro-X carboxypeptidase | 85 |
| G3IEF1 | Ferritin | 85 |
| G3IH84 | ARSA | 85 |
| G3HY95 | Transformation/transcription domain-associated protein | 61 |
| G3GY95 | Eukaryotic translation initiation factor 5A-1 | 60 |
| G3IID2 | Complement C1q tumor necrosis factor-related protein 5 | 56 |
| G3HA54 | Plasminogen activator inhibitor 1 | 44 |
| G3HTE5 | Lysosomal alpha-glucosidase | 42 |
| G3H3E4 | Galectin-3-binding protein | 40 |
| G3H9I9 | HGF/SF receptor | 38 |
| G3IAQ0 | 2-phospho-D-glycerate hydro-lyase | 16 |
| G3H8V1 | 92 kDa gelatinase | 13 |
| G3H2P3 | Beta-galactosidase (Fragment) | 13 |
| G3H170 | Drebrin | 10 |
| G3I8R9 | Endoplasmic reticulum chaperone BiP | 9 |
| G3HGQ1 | Cation-independent mannose-6-phosphate receptor | 8 |
| G3IBK2 | Filamin-A | 8 |


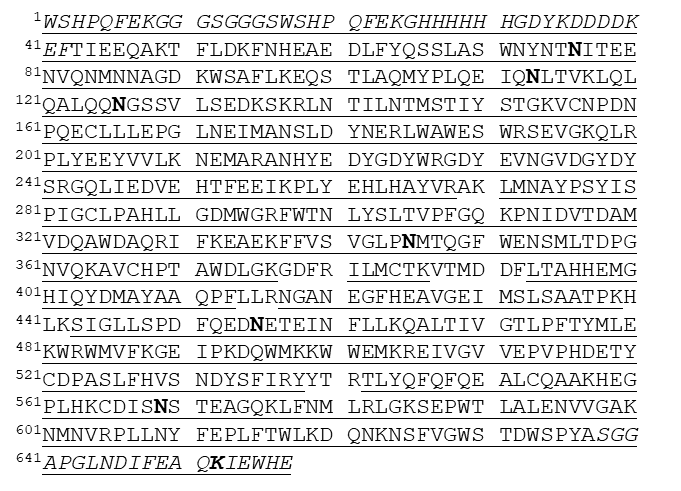


**Figure S1.** ACE2-1 LC-MS/MS-based peptide mapping from trypsin (top) and chymotrypsin (bottom) digestion. Underlined amino acids represent combined identified sequence and italics indicate strept, 6x-His, and FLAG affinity tags (N-terminus) and BirA recognition AviTag (C-terminus, biotinylated Lys residue in bold). N-glycosylation sites (Asn) shown in bold, although detected as Asp residues in deglycosylated samples.

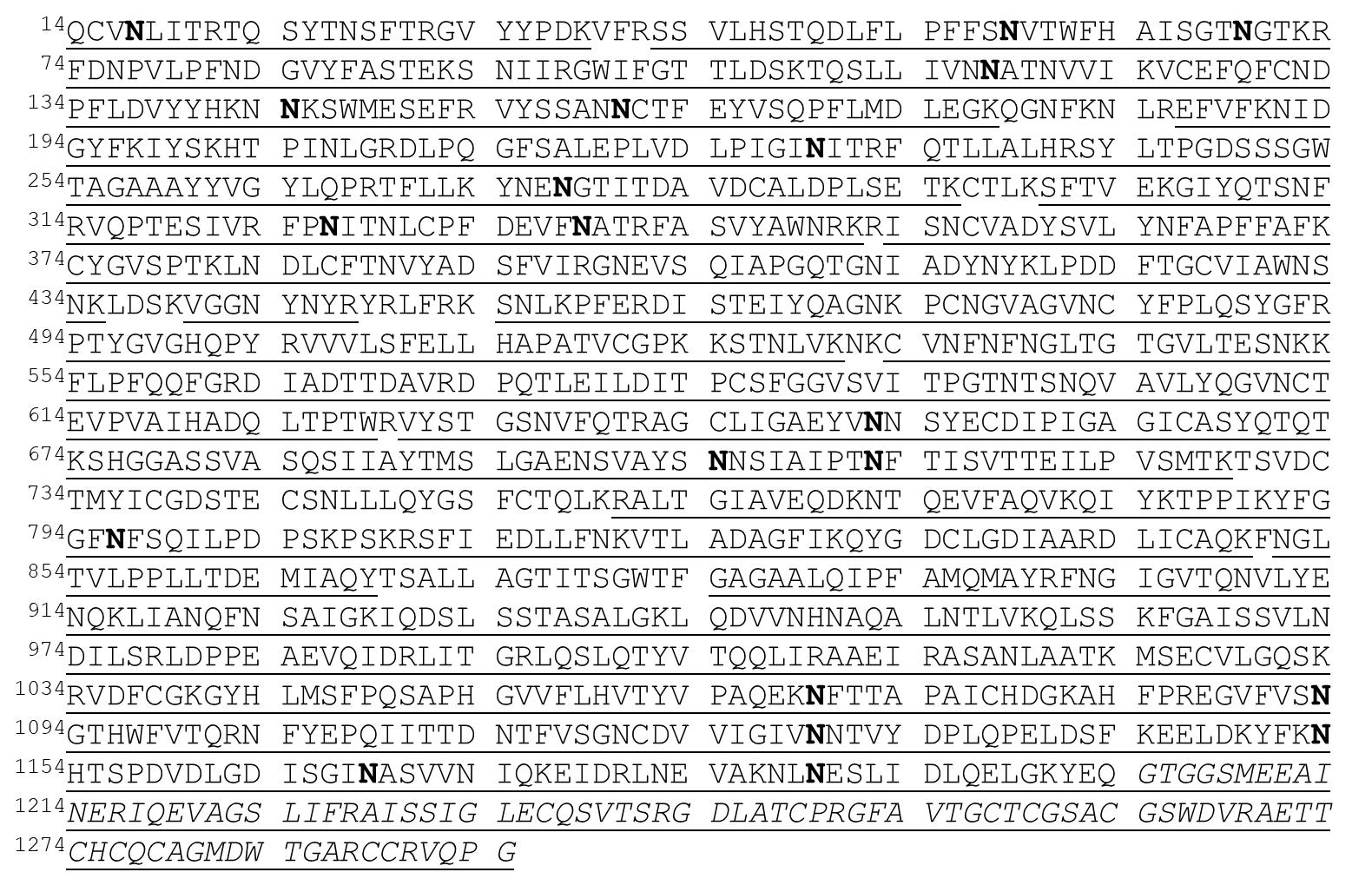


**Figure S2.** OMIC-1 LC-MS/MS-based peptide mapping from trypsin (top) and chymotrypsin (bottom) digestion. Underlined amino acids represent combined identified sequence and italics indicate the trimerization domain of human resistin (C-terminus). N-glycosylation sites (Asn) shown in bold, although detected as Asp residues in deglycosylated samples.


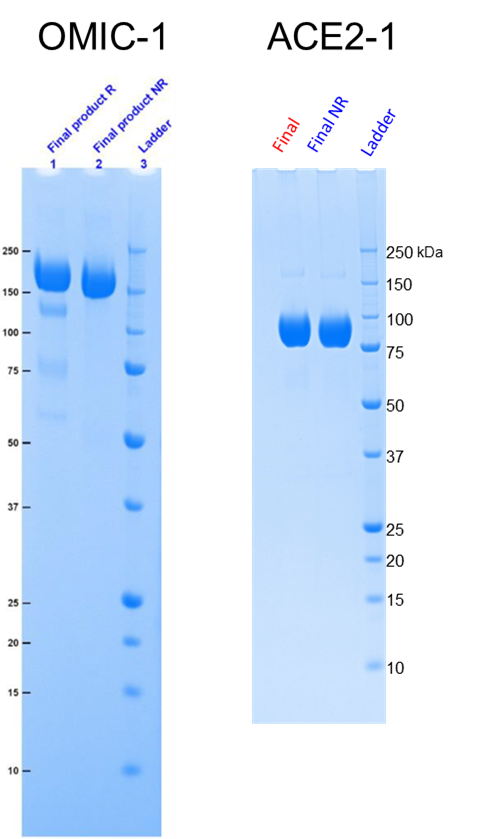


**Figure S3.** SDS-PAGE after purification of bulk protein materials used for OMIC-1 and ACE2‑1. OMIC-1 was visualized on a Novex tris-glycine 4-12 % gel, and ACE2-1 on a NuPage bis-tris 4‑12 % gel. Both gels employed MES running buffer and protein separation proceeded at 200 V for 40 min. Protein samples were prepared in both reducing (R) and non-reducing (NR) conditions.


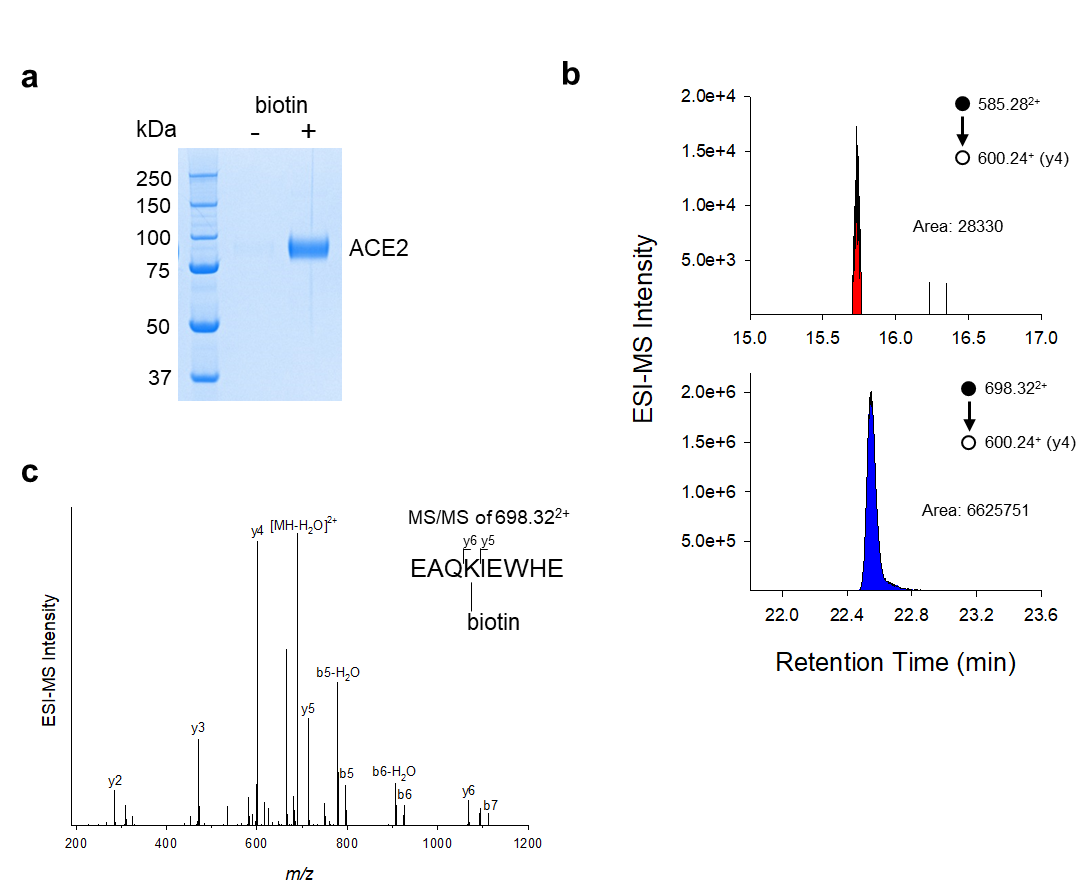


**Figure S4.** (a) Biotinylated-ACE2 interaction with immobilized avidin. Gel lanes show supernatants from washes in the absence (−) or presence (+) of supplemental biotin. (b) Parallel reaction monitoring for unmodified C-terminal ACE2 peptide (top panel, EAQKIEWHE, 585.28^2+^) and biotinylated peptide (bottom panel, 698.32^2+^). (c) MS/MS spectrum of biotinylated ACE2 C-terminal peptide with key fragment ions indicated.

**Figure S5.** LC-SEC-UV chromatograms of OMIC-1 and various dilutions prepared in formulation buffer. Inset shows zoomed view of 100X dilution.

**Figure S6.** Protein size heterogeneity determined by LC-SEC-UV. Normalized ACE2 monomer percent in ACE2-1 (a) and spike trimer percent in OMIC-1 (b). Dotted lines depict averages and dashed lines represent two standard deviations of the mean. ACE2-1 units were −80 °C controls from short-term and freeze-thaw stability studies, and OMIC-1 units were from the homogeneity study.


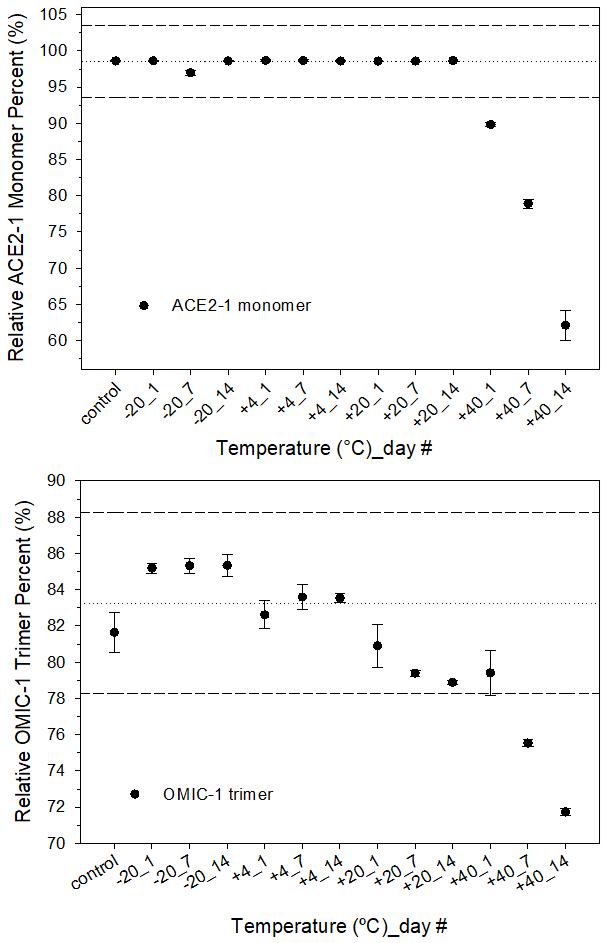


**Figure S7.** Short-term stability measurements for ACE2-1 monomer (top) and OMIC-1 trimer (bottom) determined by LC-SEC-UV. Data points are averages of duplicate units per condition, measured in triplicate and error bars represent standard deviations. Dotted lines are the averages of all −80 °C samples measured across the respective RM characterization campaigns (value assignment, short-term and freeze-thaw stability). The dashed lines show ± 5 % and are for visualization purposes only.


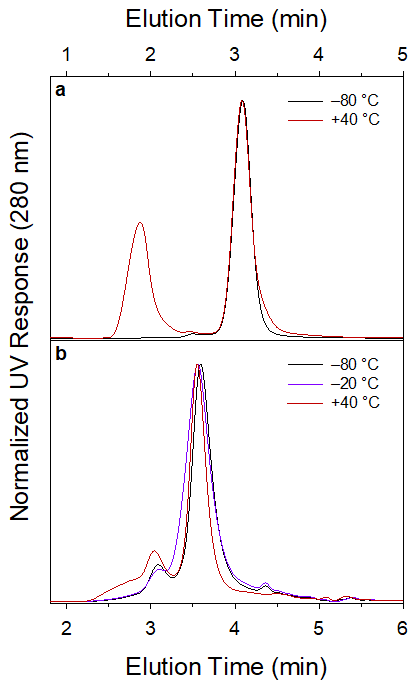


**Figure S8.** Size-exclusion UV chromatograms of (a) ACE2-1 and (b) OMIC-1 after two- week storage at −80 °C (black curves), −20 °C (blue), and +40 °C (red).


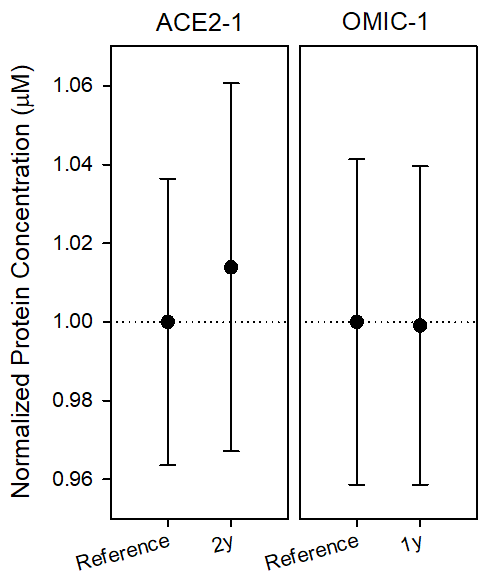


**Figure S9.** Normalized protein concentrations in ACE2-1 and OMIC-1 determined via UV-vis during value assignment (reference) and extended storage at −80 °C (y: years). Data points are the averages of triplicate measurements on at least two units per condition and error bars represent the combined standard uncertainties (*k* = 1).


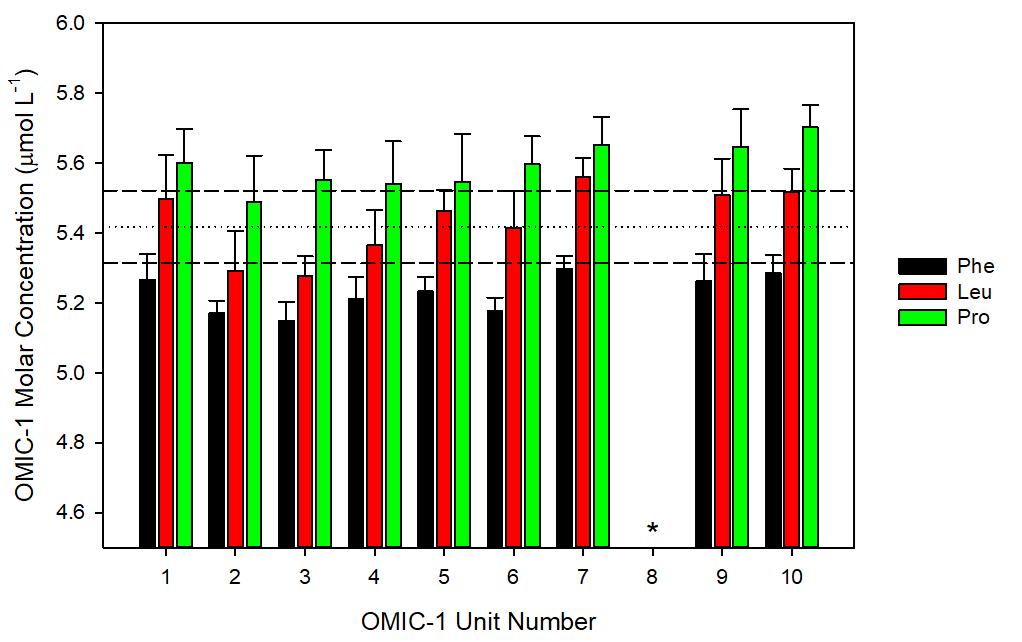


**Figure S10.** Molar protein concentration in OMIC-1 determined by amino acid analysis ID-MS after acid hydrolysis. The ten units measured are identical to those depicted in Figure 1 of the main text. The spike protein amount was determined from the mole fraction of each of the three amino acids measured (Phe, Leu, Pro) and their respective abundance in the OMIC protein sequence. Vertical bars and error bars represent averages and standard deviations, respectively, of triplicate technical replicates. Dotted line is the average protein amount determined by ID-MS and dashed lines indicate standard uncertainty (*k* = 1). No data was obtained for OMIC-1 unit 8 (asterisk) as it evaporated to dryness during hydrolysis.


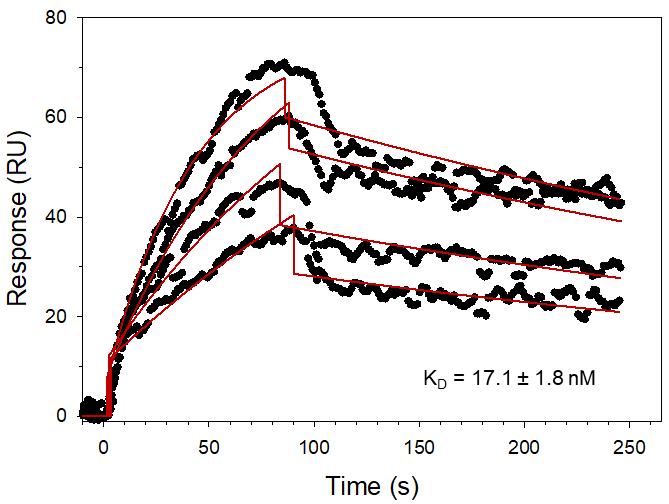


**Figure S11.** Binding affinity of SMT1-1 for immobilized ACE2-1. Spike protein concentrations covered a range of 25 – 200 nM (black circles). A 1:1 binding model was fit (red lines) to the data and the equilibrium dissociation constant represents the average and standard deviation of duplicate independent measurements.
